# A Computational Exploration of the Molecular Network Associated to Neuroinflammation in Alzheimer’s Disease

**DOI:** 10.3389/fphar.2021.630003

**Published:** 2021-07-15

**Authors:** Fatima El Idrissi, Bernard Gressier, David Devos, Karim Belarbi

**Affiliations:** ^1^ Univ. Lille, Inserm, CHU-Lille, Lille Neuroscience & Cognition, Lille, France; ^2^ Département de Pharmacologie de la Faculté de Pharmacie, Univ. Lille, Lille, France; ^3^ Département de Pharmacologie Médicale, I-SITE ULNE, LiCEND, Lille, France

**Keywords:** Alzheimer disease, cytokines, interleukins, microglia, neuroinflammation, toll-like receptors, tumor necrosis factor-alpha, neurodegenerative diseases

## Abstract

Neuroinflammation, as defined by the presence of classically activated microglia, is thought to play a key role in numerous neurodegenerative disorders such as Alzheimer’s disease. While modulating neuroinflammation could prove beneficial against neurodegeneration, identifying its most relevant biological processes and pharmacological targets remains highly challenging. In the present study, we combined text-mining, functional enrichment and protein-level functional interaction analyses to 1) identify the proteins significantly associated to neuroinflammation in Alzheimer’s disease over the scientific literature, 2) distinguish the key proteins most likely to control the neuroinflammatory processes significantly associated to Alzheimer's disease, 3) identify their regulatory microRNAs among those dysregulated in Alzheimer's disease and 4) assess their pharmacological targetability. 94 proteins were found to be significantly associated to neuroinflammation in Alzheimer’s disease over the scientific literature and IL4, IL10 and IL13 signaling as well as TLR-mediated MyD88- and TRAF6-dependent responses were their most significantly enriched biological processes. IL10, TLR4, IL6, AKT1, CRP, IL4, CXCL8, TNF-alpha, ITGAM, CCL2 and NOS3 were identified as the most potent regulators of the functional interaction network formed by these immune processes. These key proteins were indexed to be regulated by 63 microRNAs dysregulated in Alzheimer's disease, 13 long non-coding RNAs and targetable by 55 small molecules and 8 protein-based therapeutics. In conclusion, our study identifies eleven key proteins with the highest ability to control neuroinflammatory processes significantly associated to Alzheimer’s disease, as well as pharmacological compounds with single or pleiotropic actions acting on them. As such, it may facilitate the prioritization of diagnostic and target-engagement biomarkers as well as the development of effective therapeutic strategies against neuroinflammation in Alzheimer’s disease.

## Introduction

Despite extensive research there is still no cure for Alzheimer’s disease, the commonest cause of dementia. Alzheimer’s disease is characterized by neuronal loss in specific brain regions associated with the formation of amyloid-beta senile plaques and tau-immunoreactive neurofibrillary tangles (Polanco et al., 2018). Another hallmark of the disease is the presence of neuroinflammation as defined by the presence of classically activated microglial cells (Fan et al., 2017; Dani et al., 2018). Microglial cells are the resident immune cells of the central nervous system (Ransohoff and Cardona, 2010). While their activation is needed to defend the brain from injury, it has become clear that a sustained and uncontrolled inflammation could contribute to neurodegeneration, for instance leading to vascular dysfunction, mitochondrial dysfunction and oxidative stress (Sochocka et al., 2013; Orsucci et al., 2013).

Several lines of evidence support that better understanding the neuroinflammatory process in Alzheimer’s disease could lead to the development of new diagnostic and therapeutic tools. Positron emission tomography showed that microglial activation is observed from the earliest stages of Alzheimer's disease and correlates both with amyloid deposition and tau aggregation (Dani et al., 2018). Moreover, exciting data demonstrated that the immune challenges could trigger and exacerbate tau and amyloid pathologies (Bhaskar et al., 2010; Krstic et al., 2012), possibly contributing to a pathological vicious circle that could favor regulated neuronal death. Genetic studies including genome-wide studies have also associated inflammation-related genes to the etiology of Alzheimer’s disease (i.e. ABCA7, CLU, CR1, HLA-DRB1, HLA-DRB5, PICALM, and TREM2) (Lambert et al., 2009; Jun et al., 2010; Guerreiro et al., 2013; Lambert et al., 2013; Jansen et al., 2019), further supporting the concept of neuroinflammation as a pathogenic factor in Alzheimer’s disease. To date, a major challenge is to identify the key processes and proteins linked to neuroinflammation in Alzheimer’s disease. Available information reveals a complex picture with the implication of central and peripheral cell types and of a plethora of proteins including pro-inflammatory cytokines, anti-inflammatory cytokines, chemoattractant molecules, peripheral immune mediators as well as their receptors and downstream signaling targets (Itagaki et al., 1988; Togo et al., 2002; Monsonego et al., 2003). Mining useful information in these data for the development of diagnostic and therapeutic neuroprotective strategies remains highly challenging.

Integrated bioinformatics can help to explore complex biological pathways, by analyzing large amount of data and by performing analyses over many distinct datasets. In the present study, we used integrated bioinformatics to perform a systematic review of the proteins associated to neuroinflammation in Alzheimer’s disease over the scientific literature and then to identify the most represented biological processes and the key candidate proteins to modulate them. We combined text-mining, Gene Ontology and Reactome functional enrichment analysis, protein‐protein functional interaction analysis, non-coding RNA-target interaction analysis and drug–protein interaction analysis and identified eleven proteins with the highest ability to modulate neuroinflammatory processes in Alzheimer’s disease. As such, our study provides data that could help to further explore the time-dependent role of the immune response in Alzheimer’s disease and to prioritize the development of neuroinflammation-centered diagnostic or therapeutic tools against Alzheimer’s disease.

## Materials and Methods

### Protein Collection (Text-Mining)

A systematic review of the proteins relating to Alzheimer’s disease neuroinflammation was collected using the pubmed2ensembl resource that has been developed as an extension to the BioMart system for mining the biological literature for genes (http://pubmed2ensembl.ls.manchester.ac.uk/) (Baran et al., 2011). The string ‘neuroinflammation alzheimer’ was used to retrieve the Ensembl Gene ID related both to neuroinflammation and Alzheimer’s disease in the Homo sapiens (human) genome assembly GRCh37 from Genome Reference Consortium. We then used UniProtKB Retrieve/ID mapper (https://www.uniprot.org/) to retrieve the UniProtKB protein identifiers associated to these Ensembl Gene ID (UniProt, 2019). All the protein identifiers were imported into Cytoscape 3.7.2 for subsequent analysis.

### Gene Set Enrichment

Gene Ontology (GO) enrichment of the collected proteins was first performed using the ClueGo + CluePedia Cytoscape plug-in, with annotation from GO Cellular Component, GO Molecular Function and GO Biological Process categories (Bindea et al., 2009). A *p*-value <0.05 was considered as the cutoff criterion. The most enriched annotations were then visualized using the ggplot2 package in R language (www.r-project.org). Functional enrichment analysis of the proteins was subsequently performed and visualized using the Reactome Pathway database (https://reactome.org) (Fabregat et al., 2017; Jassal et al., 2020). Proteins from the 10 most significantly enriched biological processes identified with Reactome were selected for subsequent analysis.

### Protein-Protein Functional Interaction

The STRING protein query database (http://string-db.org) was used to construct a protein-protein functional interaction network in Cytoscape (Szklarczyk et al., 2019). The minimum required interaction score was set to 0.7 to retain only high-confidence functional interactions. We then used the CentiScaPe Cytoscape plug-in to calculate the node degree and betweenness centrality of each protein. The nodes that had a degree centrality and a betweenness centrality greater than or equal to the mean were identified as key proteins (e.g. more likely to modulate neuroinflammation) and retained for subsequent microRNA-target and drug-protein interaction analyses.

### Integration of Regulatory MicroRNAs and Long Non-Coding RNAs (lncRNAs) in the Key Protein Network

The microRNA-target interactions were identified using the miRTarBase Homo sapiens 8.0 database that curates experimentally validated microRNA-target interactions and includes 502,652 microRNA target interactions (Huang et al., 2020). The lncRNA-target interactions were obtained from the LncRNA2Target 2.0 database, by retaining only interactions inferred from the low-throughput experiments such as immunoprecipitation assays, RNA pull down assays, luciferase reporter assays, RT-qPCR or western blot (Cheng et al., 2019). Regulating microRNAs that are significantly dysregulated in the blood of Alzheimer's disease patients were selected based on the data published by Leidinger and coworkers (Additional file 1 of their electronic Supplementary Material; (Leidinger et al., 2013). The microRNA-target and lncRNA-target interactions and the microRNAs relative abundance in Alzheimer's disease (log2(fold change); (Leidinger et al., 2013) were mapped using the CyTargetLinker v4.1.0 Cytoscape application (Kutmon et al., 2018).

### Drug-Gene Interaction Analysis

The Drug–Gene Interaction database 3.0 (DGIdb v3.0; http://www.dgidb.org/) was used to search for existing associations between drugs (e.g. small molecule compounds or immunotherapies) and essential proteins from our dataset (Cotto et al., 2018). The results of the search were visualized using Cytoscape so that information on protein targetability, drug-protein interaction, drug nature and drug FDA-approval could be easily apprehended.

## Results

### Identification of Proteins Associated to Alzheimer’s Neuroinflammation

**FIGURE 1:**
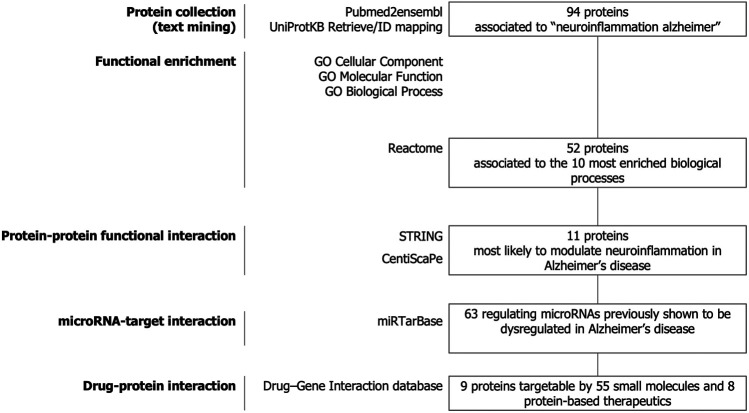
Study design. Text-mining was used to support large-scale screening of the scientific literature to collect 94 proteins related to neuroinflammation in Alzheimer’s disease. The entire dataset was analyzed for functional enrichment by using Gene Ontology (GO) cellular localization, molecular functions, biological pathways and by using the Reactome Pathway database and 52 proteins were associated to the 10 most significantly enriched pathways. Protein-protein functional interaction using STRING and CentisScaPe allowed us to identify 11 key proteins most likely to modulate neuroinflammation in Alzheimer’s disease. Finally, non-coding RNA-target interaction and drug-gene interaction analysis showed that the key proteins were indexed to be targetable by a total of 63 microRNAs dysregulated in Alzheimer's disease and by 55 small molecules and 8 protein-based therapeutics.

The strategy of our study to identify key candidate proteins to modulate neuroinflammation in Alzheimer’s disease is depicted in Figure 1. Text-mining using the pubmed2ensembl resource that links over 2,000,000 articles to nearly 150,000 genes (Baran et al., 2011) allowed us to systematically collect a list of 94 unique proteins related to neuroinflammation in Alzheimer’s disease. The list of the 94 proteins with corresponding Gene symbols, Ensembl Gene and UniProtKB identifiers is provided in Supplementary Table 1.

**FIGURE 2:**
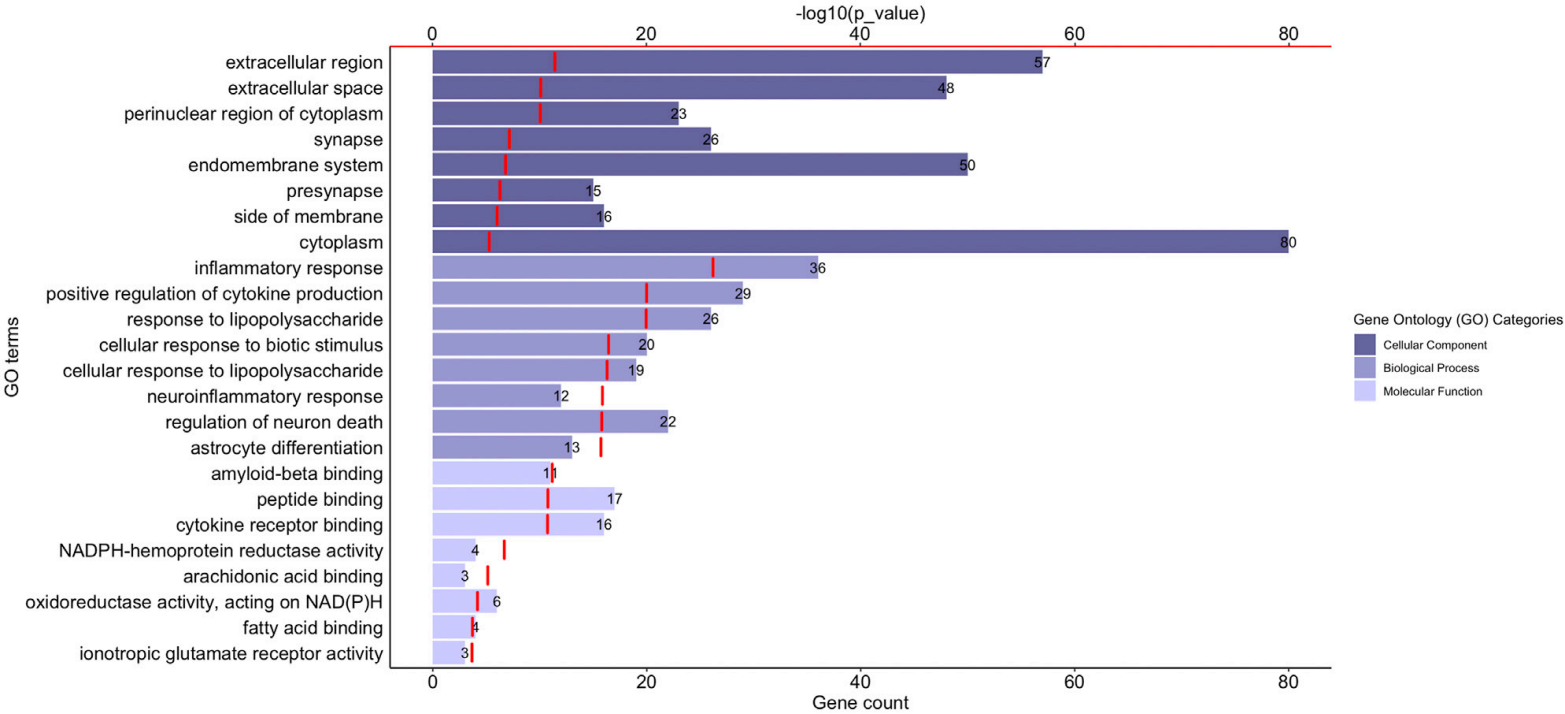
Functional enrichment analysis of the proteins associated to neuroinflammation in Alzheimer’s disease. The graph shows the top eight over-represented Gene Ontology (GO) cellular localization, GO molecular functions and GO biological pathways annotations (*Y*-axis), the number of proteins of our dataset included in each term (length of the histogram bars and number displayed next to each histogram bar, lower *X*-axis) and the −log10(*p*_value) levels (red segments, upper *X*-axis). *p*-value ≤ 0.05.

**FIGURE 3:**
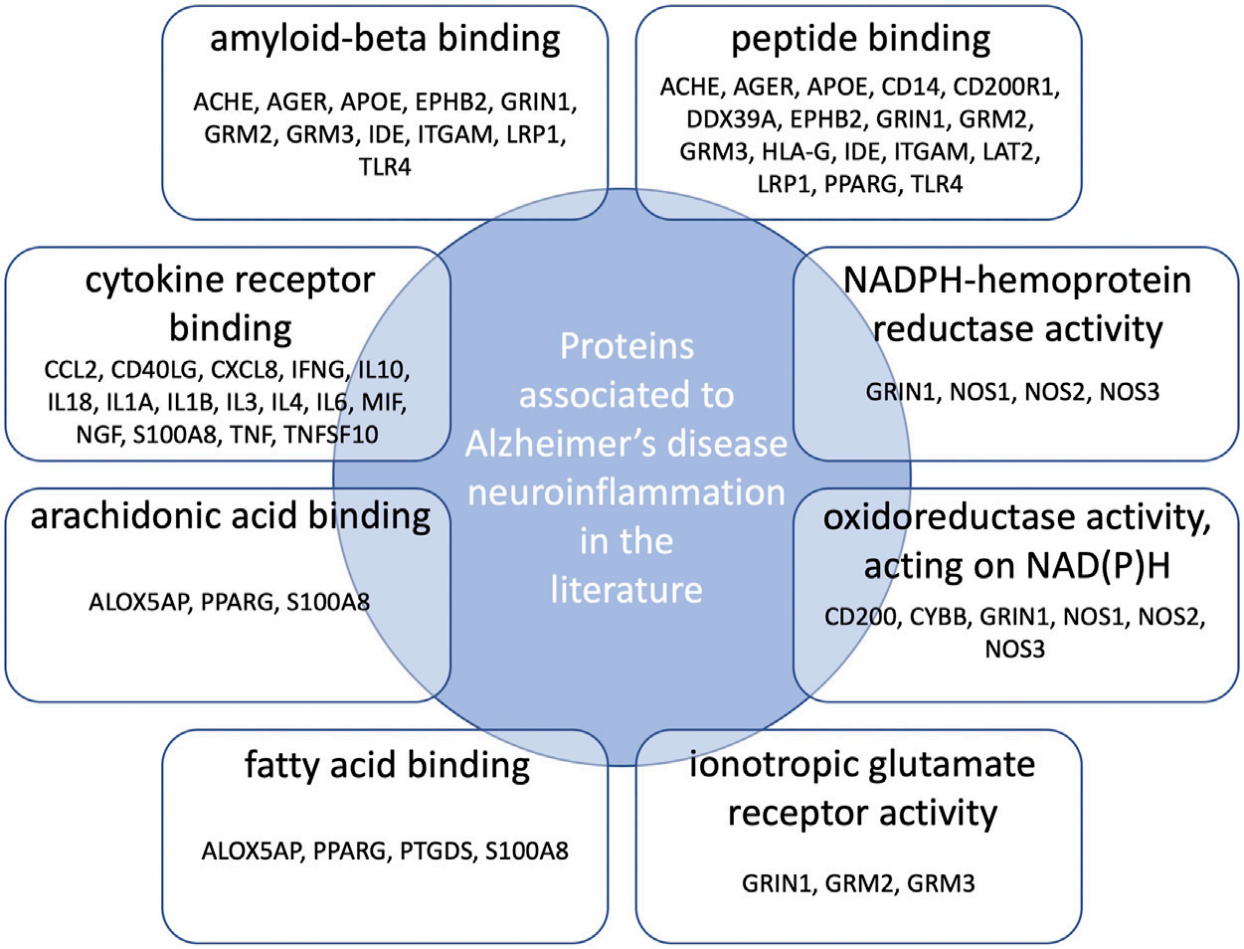
Connections between proteins associated to neuroinflammation in Alzheimer’s disease and the top eight over-represented molecular functions annotations. Protein names are those belonging both to our dataset and the enriched GO molecular functions annotations.

Gene Ontology (GO) functional enrichment analyses were performed using ClueGo + CluePedia plug-in to explore the cellular localization, molecular functions and biological pathways associated to these proteins. All the collected genes had functional annotations in the selected ontologies. The Cellular Component annotations showed an important enrichment (e.g. over-representation) of the proteins located in the extracellular region, at the cell surface or periphery or at the synapse. The gene ontology enrichment analysis of Biological process annotations showed that the most enriched terms were *inflammatory response*, *positive regulation of cytokine production* and *response to lipopolysaccharide*. Finally, Molecular function annotations revealed an enrichment for proteins with *amyloid-beta or peptide binding* activities or *cytokine receptor binding activities*. Other enriched terms were related to arachidonic acid and fatty acid binding, to NADPH oxidoreductase activity or to ionotropic glutamate receptor activity. The top eight enriched terms of the Cellular Component, Biological Process and Molecular Function are shown in Figure 2. The proteins of our dataset that are annotated for the top eight enriched Molecular Function annotations are listed in Figure 3.

Together, our results show that the proteins associated to Alzheimer's disease neuroinflammation are primarily connected to amyloid beta as well as oxidative stress and lipid metabolism that are critically involved in the neuropathogenesis of Alzheimer's disease.

### Selection of the Most Relevant Biological Processes

To identify the most relevant biological processes involved in neuroinflammation in Alzheimer’s disease, we performed a functional enrichment of the collected proteins by using the Reactome Pathway database. The 10 most relevant pathways sorted by *p*-value were *Interleukin-4 and Interleukin-13 signaling* (*p* = 1.11 × 10^−16^), *Signaling by Interleukins* (*p* = 1.11 × 10^−16^), *Interleukin-10 signaling* (*p* = 5.77 × 10^−15^)*, Immune System* (*p =* 9.30 × 10^−14^), *Cytokine Signaling in Immune system* (*p* = 5.70 × 10^−13^), *Innate Immune System* (*p* = 6.60 × 10^−8^), *Toll-like Receptor Cascades* (*p* = 8.11 × 10^−7^), *TRAF6 mediated induction of NFkB and MAP kinases upon TLR7/8 or 9 activation* (*p* = 1.00 × 10^−6^), *MyD88 dependent cascade initiated on endosome* (*p* = 1.085 × 10^−6^) and *Toll Like Receptor 7/8 (TLR7/8) Cascade* (*p* = 1.085 × 10^−6^) (Table 1). 52 proteins from the 10 aforementioned most enriched pathways were retained for protein-protein functional interaction analysis.

**TABLE 1:** Function enrichment results of proteins associated to neuroinflammation in Alzheimer’s disease using the Reactome Pathway database. Count: enriched gene number in the category.

Term	Count	*p*-value
Interleukin-4 and Interleukin-13 signaling	17	1.11 × 10^−16^
Signaling by interleukins	29	1.11 × 10^−16^
Interleukin-10 signaling	12	5.77 × 10^−15^
Immune system	53	9.30 × 10^−14^
Cytokine signaling in immune system	33	5.70 × 10^−13^
Innate immune system	29	6.60 × 10^−8^
Toll-like receptor cascades	10	8.11 × 10^−7^
TRAF6 mediated induction of NFkB and MAP kinases upon TLR7/8 or 9 activation	8	1.00 × 10^−6^
MyD88 dependent cascade initiated on endosome	8	1.085 × 10^−6^
Toll like receptor 7/8 (TLR7/8) cascade	8	1.085 × 10^−6^

### Identification of High-Confidence Protein Functional Interactions

**FIGURE 4:**
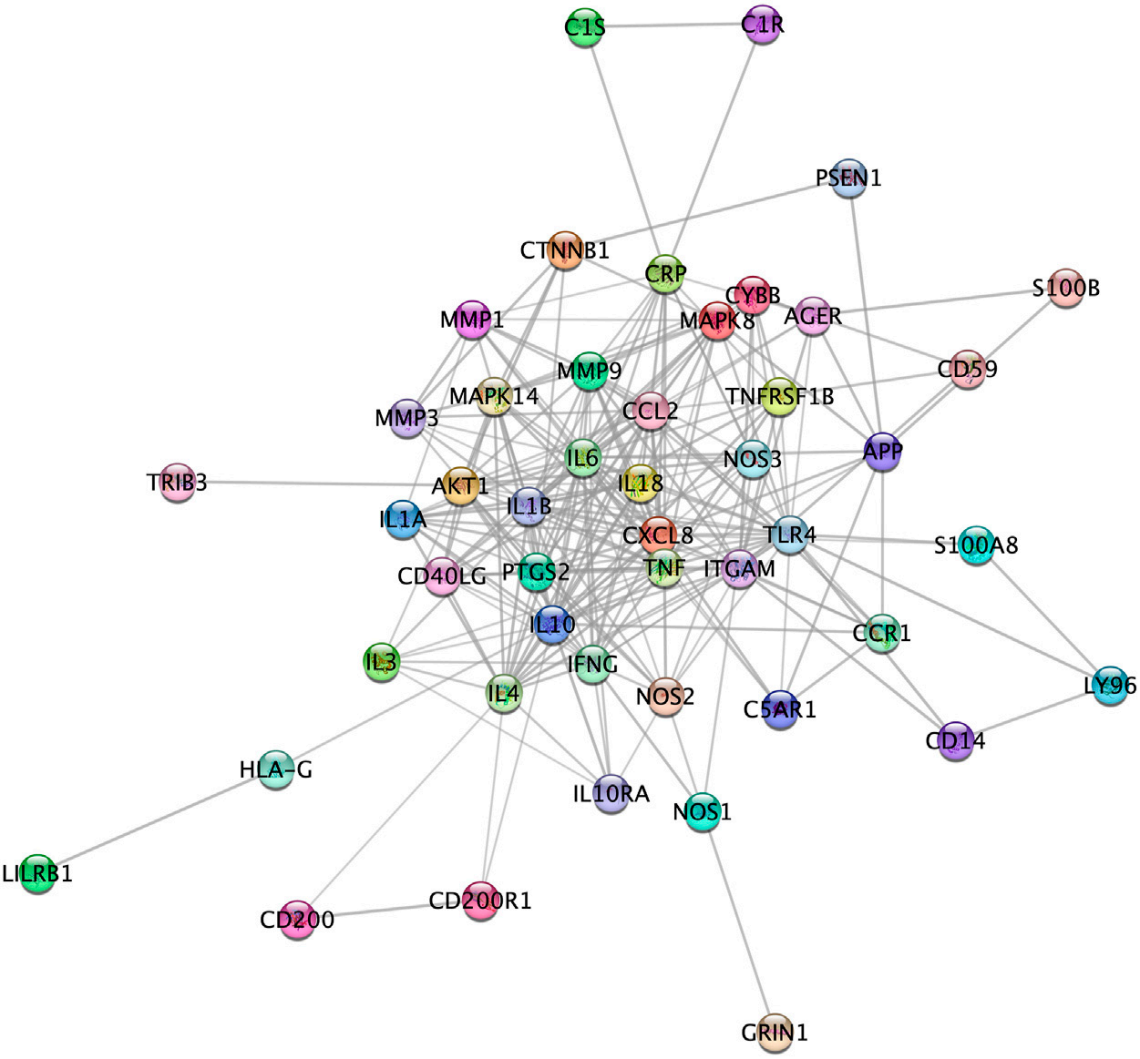
High confidence protein-protein functional interaction network of proteins associated to the most relevant biological processes in neuroinflammation in Alzheimer’s disease. Among the 52 proteins associated to the biological processes, 5 (CD4, CHGA, GIG25, LAT2 and MIF) did not show high confidence protein-protein functional interaction in the network and therefore are not represented here.

Figure 4 represents the protein-protein functional interaction network of the aforesaid proteins. In this representation, nodes represent proteins and edges represent functional interactions. There were 47 interconnected nodes (e.g. proteins) and 244 relationship pairs (e.g. functional interactions). Degree average value was 9.38 and betweenness average value was 47.69. We selected nodes with both higher than average degree (e.g. the measure of the total number of edges connected to the protein) and betweenness (e.g. an indicator of how important the protein is to the shortest paths through the network) as key proteins most likely to have the ability to modulate the aforementioned biological processes and thus neuroinflammation in Alzheimer’s disease. The 11 proteins that met this selection criterion sorted by high to low betweenness value were: interleukin (IL)10, Toll-like receptor (TLR)4, IL6, AKT1, C-reactive protein (CRP), IL4, C-X-C motif chemokine ligand (CXCL)8, Tumor necrosis factor (TNF) alpha, integrin alpha M (ITGAM), C-C motif chemokine ligand (CCL)2 and nitric oxide synthase (NOS)3 (Table 2). These proteins should be considered as the most potent modulators of neuroinflammation in Alzheimer’s disease and were next analyzed for microRNA- and drug-protein interaction.

**TABLE 2:** Proteins with highest than average betweenness and degree in the protein-protein functional interaction network.

Protein name	UniProtKB ID	Betweenness	Degree
IL10	P22301	300,549477	24
TLR4	O00206	253,174429	24
IL6	P05231	208,838661	29
AKT1	P31749	204,665414	17
CRP	P02741	190,958865	15
IL4	P05112	127,805378	18
CXCL8	P10145	118,536469	22
TNF	P01375	116,879025	25
ITGAM	P11215	94,1162934	17
CCL2	P13500	70,1571213	22
NOS3	P29474	62,2581681	11

### Integration of Regulatory Non-Coding RNAs Into the Key Protein Network

**FIGURE 5:**
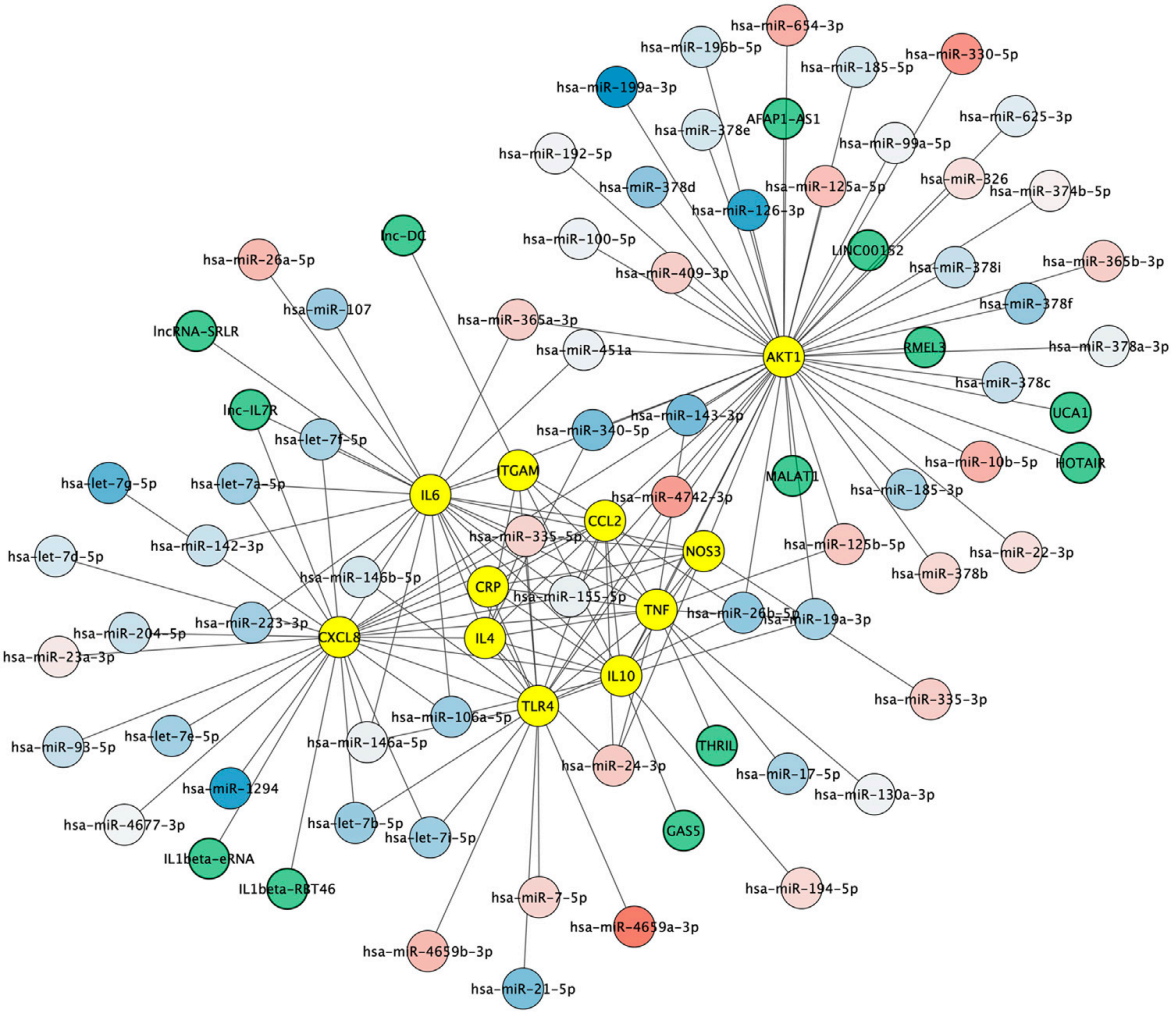
Non-coding RNA-protein interaction network. Experimentally validated interactions between microRNAs dysregulated in Alzheimer (blue to red round shapes), lncRNAs (green round shapes) and key proteins in Alzheimer’s disease neuroinflammation (yellow round shapes). Blue to red gradient denotes low to high relative abundance of microRNAs in Alzheimer's disease patient samples according to (Leidinger et al., 2013).

Non-coding RNAs including microRNAs and lncRNAs play crucial roles in regulating gene expression and have emerged as key regulators of immune cell functions in innate and adaptive immunity. We first aimed to identify microRNAs both 1) regulating the key protein network as indexed in the experimentally validated gene–microRNA interaction database miRTarBase and 2) dysregulated in Alzheimer's disease blood samples, as reported by Leidiger and coworkers (Leidinger et al., 2013). This led to the identification of 63 dysregulated mature microRNAs including 22 with higher and 41 with lower expression levels in Alzheimer's disease (Figure 5). The corresponding list of mature microRNAs with protein targets is provided in Supplementary Table 2. We also integrated 13 lncRNAs known to regulate the key protein as indexed in the LncRNA2Target database (Figure 5). The corresponding list of lncRNAs with protein targets and bibliographic references is provided in Supplementary Table 3.

### Drug–Protein Network Construction and Identification of Targetable Proteins

**FIGURE 6:**
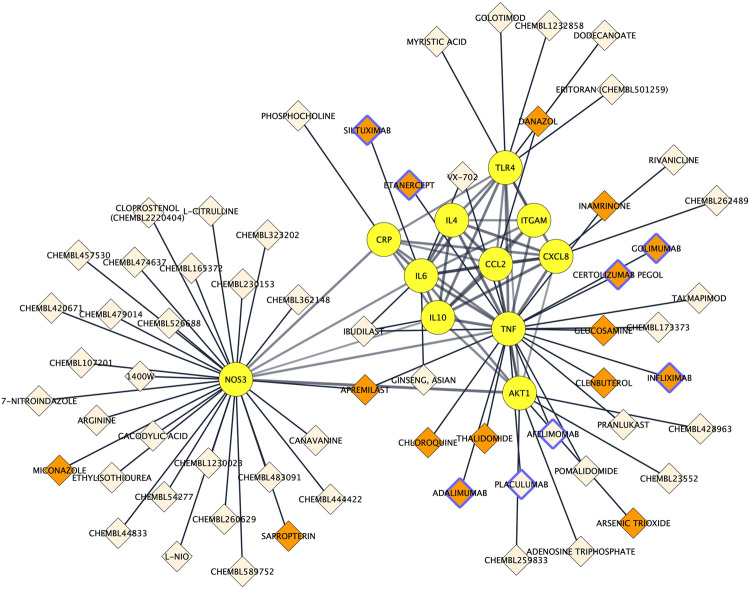
Protein-drug interaction network. Existing associations between drugs (e.g. small molecule compounds or immunotherapies; diamond shapes) and key proteins in Alzheimer’s disease neuroinflammation (yellow round shapes). FDA‐approved drugs and non‐FDA‐approved drugs are respectively colored in orange and light beige. Protein-based therapies are bordered with purple.

To assess to what extent the 11 proteins could be pharmacologically targeted, drug-proteins relationships were predicted using the Drug–Gene Interaction database 3.0. Among the 11 proteins, 9 were indexed to be targeted by a total of 63 drugs. The NOS3 protein had the most interactions with drugs (*n* = 29), followed by TNF alpha (*n* = 19), AKT1 (*n* = 5) and TLR4 (*n* = 5) and IL6 (*n* = 4). There was a total of 67 drug–protein relationships in the network. Drugs comprised 8 protein-based therapies of which 6 had a FDA-approval as well as 55 small compounds of which 10 had a FDA-approval. Drug-protein interactions can be visualized in Figure 6.

## Discussion

This study used text-mining and data-based bioinformatics to explore the protein network underlying neuroinflammatory processes in Alzheimer’s disease. Text-mining has been recommended for large-scale screening (O'Mara-Eves et al., 2015) and can be used to identify key pathways and candidate genes for drug-discovery and Human health (Singhal et al., 2016; Lever et al., 2019; Pan et al., 2019). In our study, it allowed to collect 94 proteins significantly associated to neuroinflammation in Alzheimer’s disease over the scientific literature (Supplementary Table 1). It is important to note that text-mining has biases due to the imbalance in research attention where some genes are highly investigated while others are ignored (Wang et al., 2015; Oprea et al., 2018). Therefore, the obtained list of proteins is not meant to be exhaustive and may particularly miss proteins still sparsely mentioned in the literature. As such our study may be considered as complementary to other studies such as genome-wide association studies for the further development of diagnostic and target-engagement biomarkers for drug discovery.

Gene Ontology enrichment analysis was used to get first insights into the profile of the 94 collected proteins (Bindea et al., 2009). Cellular localization annotations showed an overrepresentation of cellular compartments essential for communication (e.g. *extracellular space*, *synapse*, *presynaptic compartment* and *side of membranes*) consistent with the fact that an altered cerebral microenvironment and a dysregulated cell-to-cell crosstalk are critically involved in neuroinflammation (Schwartz and Deczkowska, 2016; Fabiani and Antollini, 2019). Most overrepresented molecular function annotations were *amyloid beta binding activity*—an activity presented by proteins such as ITGAM (also named CD11b) (Goodwin et al., 1997) and TLR4 (Liu et al., 2020)—as well as *cytokine receptor binding activity*, further linking the amyloid peptide to the innate immune response in Alzheimer's disease. Molecular function annotations also defined NADPH oxidation, *fatty acid* and *arachidonic acid binding* as well as *glutamate signaling* as significant functions, therefore connecting neuroinflammation to NADPH oxidases and arachidonic acid metabolism critically involved in the neuropathogenesis of Alzheimer's disease (Fricker et al., 2018; Tarafdar and Pula, 2018; Chen et al., 2020). Altogether, the Gene Ontology annotation enrichment helped to capture a coherent picture of neuroinflammation in Alzheimer’s disease and its connections to relevant molecular functions and biological pathways.

Finding the biological processes that are mostly associated to neuroinflammation in Alzheimer’s disease remains highly challenging. We aimed to address this challenge without *a priori* by analyzing the functional enrichment of our protein dataset using the Reactome Pathway database. Signaling of the anti-inflammatory cytokines IL4, IL10 and IL13 appeared as the most significantly enriched processes. These pathways have been proposed to have protective role in Alzheimer's disease by inducing the clearance of amyloid-beta and the secretion of anti-inflammatory cytokines and neurotrophic factors (Szczepanik et al., 2001; Kawahara et al., 2012; Kiyota et al., 2012; Tang et al., 2019). On the opposite, IL4 and IL13 signaling could exacerbate oxidative stress by activating microglial NADPH oxidases and cyclooxygenase 2 (Park et al., 2009; Jeong et al., 2019). Likewise, TLR signaling as well as the MyD88 and the TNF receptor associated factor (TRAF)6 dependent molecular cascades (that both play an essential role in TLR-elicited intracellular signaling) were identified as important processes. Here again, both beneficial and detrimental roles were attributed to TLRs and for instance TLR7, TLR8 and TLR9 signaling could enhance microglial amyloid beta uptake in the early stage of Alzheimer’s disease, but over time contribute to sustained neuroinflammation (Gambuzza et al., 2014). Taken together, our data highlight that the main processes associated to neuroinflammation in Alzheimer’s disease have both beneficial as well as detrimental roles, and these may be time-dependent. As such, developing strategies to enhance their protective effects or to combat their pathological responses in a disease-stage manner could prove therapeutic potential for Alzheimer’s disease.

Identifying the proteins critically involved in the regulation of the aforementioned processes required to detect key proteins based on the network topology. We found 11 proteins with higher than average degree and betweenness and thus with the highest ability to control the immune processes. These key proteins included the anti-inflammatory cytokines IL4 and IL10 and the pro-inflammatory cytokines TNF-alpha and IL6 that balance appears to be important for maintaining a less pathologic immune profile, as suggested by a recent study of Taipa and coworkers that found that a collection of both pro-inflammatory and anti-inflammatory cytokines were correlated with less cognitive decline in patients after one year (Taipa et al., 2019). Key proteins also included the neutrophil chemoattractant CXCL8 and CCL2 (also named monocyte chemoattractant protein (MCP)1) that exert their effects on target cells via the G protein-coupled receptors CXCL5/CXCL8 receptor (CXCR)1 and 2 and the C-C chemokine receptor (CCR)2, respectively (Zhang et al., 2013; Mamik and Ghorpade, 2016; Chou et al., 2018; Haarmann et al., 2019). Of note, both CXCL8 and CCL2 levels were found to be increased in the cerebrospinal fluid and brain tissue of Alzheimer’s disease patients (Galimberti et al., 2006; Sokolova et al., 2009) and higher CCL2 levels in cerebrospinal fluid were associated with a faster rate of cognitive decline during the early stages of the disease (Westin et al., 2012). Our study also pinpointed at TLR4 and ITGAM (also named CD11b, one protein subunit that forms macrophage antigen complex-1 (Mac-1) or complement receptor 3 (CR3)) that stimulation by amyloid beta leads to microglia activation, resulting in increased cytokine production and oxidative stress (Hong et al., 2016; Yang et al., 2020). Levels of natural CR3 ligands, such as complement fragments, ICAM-1, and fibrin, are also increased in Alzheimer's disease patients (van Oijen et al., 2005; Daborg et al., 2012; Janelidze et al., 2018). Of interest, the activation of CCR2 (Bakos et al., 2017), CXCR1 and CXCR2 (Lane et al., 2006), TLR4 (Fang et al., 2017) and ITGAM (Zhang et al., 2011) all trigger the phosphoinositide 3-kinase (PI3K)/Akt pathway that is perturbated in the brain of patients (Steen et al., 2005; Liu et al., 2011) and that downstream targets include the kinase AKT1, another key protein in our results. Also of interest, the activity of calcium-responsive NOS3, another key node in our study otherwise known as an important regulator of vascular function and of oxidative homeostasis, is also intertwined with that of AKT1 (Dossumbekova et al., 2008; Silva and Garvin, 2009). Finally, our study identified CRP that is increased in brain tissue from Alzheimer’s disease patients (Wood et al., 1993; Iwamoto et al., 1994) and that should be considered not only as a marker but also as a driver of neuroinflammation, since CRP can bind to the complement factor 1q (C1q) and activate the classical complement cascade (Slevin et al., 2015; Braig et al., 2017). The identification of these 11 functionally interconnected proteins provides more specific biomarkers modulating neuroinflammatory processes in Alzheimer's disease. Furthermore, the identification of microRNAs regulating these proteins and known to be deregulated in Alzheimer's disease such as hsa-miR-26a-5p, hsa-miR-107, hsa-miR-26b-5p or hsa-let-7f-5p support their implication in Alzheimer's disease pathogenesis as well as their interest as potential peripheral biomarkers (Wang et al., 2008; Mendes-Silva et al., 2016; Guo et al., 2017; Swarbrick et al., 2019). Likewise, the identification of lncRNAs acting on targets such as CXCL8, TNF-alpha, IL6, IL10 or ITGAM support their role, still largely unexplored, as important regulators of the human innate immune response (Wang et al., 2014a; Cui et al., 2014; Ilott et al., 2014; Li et al., 2014; Li et al., 2017) (Supplementary Table 3).

Drug–protein interactions analysis revealed that 9 of the 11 key proteins could be targeted by a total of 55 small molecules and 8 protein-based therapeutics. The latter comprised FDA approved TNF-alpha antagonists (infliximab, etanercept, adalimumab, golimumab and certolizumab pegol) that were found to be associated with lower Alzheimer’s disease risk in patients with rheumatoid arthritis and psoriasis (Zhou et al., 2020) and proved positive immune and cognitive outcomes in rodent models of Alzheimer’s disease (Kim et al., 2016; Paouri et al., 2017; Park et al., 2019). They also included the IL6-targeting antibody siltuximab that was launched for the treatment of multicentric Castleman but that has not been evaluated to date in the context of Alzheimer’s disease, to our knowledge. Drug-protein interaction analysis also pictured small molecules such as thalidomide that reduces the production of TNF-alpha (Sampaio et al., 1991; Gabbita et al., 2012) and that more potent derivative 3,6-dithiothalidomide ameliorated cognition in a rat models of LPS-induced sustained microglia activation (Belarbi et al., 2012) and in a triple transgenic mouse model of Alzheimer's disease (Tweedie et al., 2012). Likewise, we identified glucosamine that was shown to inhibit in microglial cells lipopolysaccharide-induced TNF-alpha expression, Ca2+ influx and outward K+ currents, which are typically representative of microglial activation (Yi et al., 2005). Interestingly, our analysis also highlighted several phosphodiesterase inhibitors such as apremilast (phosphodiesterase 4 inhibitor) that interferes with NOS3 and cytokines production (Schett et al., 2010; Gulisano et al., 2018), inamrinone (phosphodiesterase 3 inhibitor) or ibudilast (a non-selective 3, 4, 10, 11 phosphodiesterase inhibitor). Of note, ibudilast is currently approved for use as an anti-inflammatory in Japan, improved amyloid beta-induced cognitive impairment in rodent (Wang et al., 2014b) and acts through TLR4 blockade (Schwenkgrub et al., 2017a), reduction of pro-inflammatory cytokines TNF-alpha, IL6 and IL1-beta (Wakita et al., 2003; Schwenkgrub et al., 2017b), up-regulation the anti-inflammatory cytokine IL4 and IL10 and various neurotrophic factors (Mizuno et al., 2004). Although these drug-protein association should overall be taken with caution, given that the drugs' effects on the network may depend on their dose and on the disease stage and severity, they may be useful to explore the neuroinflammatory cascade involved in Alzheimer’s disease.

In conclusion, our study provides a review of proteins associated to neuroinflammation in Alzheimer’s disease and identifies 11 interrelated key proteins with the highest ability to control neuroinflammatory processes in Alzheimer’s disease. While some proteins such as TNF-alpha are largely evaluated, our data provide evidence to encourage further investigation on others such as CCL2, CXCL8, IL-10, TLR4, CRP or AKT1 that appears as a key component for translating extracellular information into downstream biological responses regulating microglia phenotype. Assessing these proteins could then help for the evaluation of disease-modifying properties, for example for assessing the impact of the recently FDA-approved aducanumab on neuroinflammation-related endpoints. A major challenge for future therapies will be to enhance the protective role or combat the pathological roles of immune processes such as IL4, IL10 and IL13 or TLR-mediated MyD88- and TRAF6- dependent signaling, and this will require to better understanding the time-dependent role of these processes in Alzheimer’s disease. Our study prioritizes biomarkers and putative targets and identifies non-coding RNAs and pharmacological compounds with single or pleiotropic actions acting on them. As such, it may facilitate the way to novel diagnostic and therapeutic neuroprotective strategies for Alzheimer’s disease.

## Data Availability

The original contributions presented in the study are included in the article/Supplementary Material, further inquiries can be directed to the corresponding author.
