# Supplementary material for: A Computational Exploration of the Molecular Network Associated to Neuroinflammation in Alzheimer’s Disease
Source: Front Pharmacol. 2021 Jul 15;12:630003. doi: 10.3389/fphar.2021.630003 (PMC8319636; doi:10.3389/fphar.2021.630003)
Supplement: Supplementary file 1 [file Table1.DOCX]

**Supplementary table 1. Proteins associated to Alzheimer’s disease neuroinflammation collected using the pubmed2ensembl resource.**

| **Ensembl Gene ID** | **UniProtKB ID** | **Gene symbols** | **Protein name** |
| --- | --- | --- | --- |
| ENSG00000175899 | P01023 | A2M CPAMD5 FWP007 | Alpha-2-macroglobulin |
| ENSG00000087085 | P22303 | ACHE | Acetylcholinesterase ( |
| ENSG00000204305,  ENSG00000206320,  ENSG00000234729,  ENSG00000237405,  ENSG00000229058 | Q15109 | AGER RAGE | Advanced glycosylation end product-specific receptor |
| ENSG00000159723 | O00253 | AGRP AGRT ART | Agouti-related protein |
| ENSG00000142208 | P31749 | AKT1 PKB RAC | RAC-alpha serine/threonine-protein kinase |
| ENSG00000132965 | P20292 | ALOX5AP FLAP | Arachidonate 5-lipoxygenase-activating protein |
| ENSG00000130203 | P02649 | APOE | Apolipoprotein E |
| ENSG00000142192 | P05067 | APP A4 AD1 | Amyloid-beta precursor protein |
| ENSG00000176697 | P23560 | BDNF | Brain-derived neurotrophic factor |
| ENSG00000159403 | P00736 | C1R | Complement C1r subcomponent |
| ENSG00000182326 | P09871 | C1S | Complement C1s subcomponent |
| ENSG00000197405 | P21730 | C5AR1 C5AR C5R1 | C5a anaphylatoxin chemotactic receptor 1 |
| ENSG00000108691 | P13500 | CCL2 MCP1 SCYA2 | C-C motif chemokine 2 |
| ENSG00000118523 | P29279 | CCN2 CTGF HCS24 IGFBP8 | CCN family member 2 |
| ENSG00000163823 | P32246 | CCR1 CMKBR1 CMKR1 SCYAR1 | C-C chemokine receptor type 1 |
| ENSG00000170458 | P08571 | CD14 | Monocyte differentiation antigen CD14 |
| ENSG00000091972 | P41217 | CD200 MOX1 MOX2 My033 | OX-2 membrane glycoprotein |
| ENSG00000163606 | Q8TD46 | CD200R1 CD200R CRTR2 MOX2R OX2R UNQ2522/PRO6015 | Cell surface glycoprotein CD200 receptor 1 |
| ENSG00000010610 | P01730 | CD4 | T-cell surface glycoprotein CD4 |
| ENSG00000102245 | P29965 | CD40LG CD40L TNFSF5 TRAP | CD40 ligand |
| ENSG00000085063 | P13987 | CD59 MIC11 MIN1 MIN2 MIN3 MSK21 | CD59 glycoprotein |
| ENSG00000100604 | P10645 | CHGA | Chromogranin-A |
| ENSG00000132693 | P02741 | CRP PTX1 | C-reactive protein |
| ENSG00000168036 | P35222 | CTNNB1 CTNNB OK/SW-cl.35 PRO2286 | Catenin beta-1 |
| ENSG00000169429 | P10145 | CXCL8 IL8 | Interleukin-8 |
| ENSG00000121966 | P61073 | CXCR4 | C-X-C chemokine receptor type 4 |
| ENSG00000165168 | P04839 | CYBB NOX2 | Cytochrome b-245 heavy chain |
| ENSG00000123136 | O00148 | DDX39A DDX39 | ATP-dependent RNA helicase DDX39A |
| ENSG00000104325 | Q16698 | DECR1 DECR SDR18C1 | 2,4-dienoyl-CoA reductase, mitochondrial |
| ENSG00000133216 | P29323 | EPHB2 DRT EPHT3 EPTH3 ERK HEK5 TYRO5 | Ephrin type-B receptor 2 |
| ENSG00000149564 | Q96AP7 | ESAM UNQ220/PRO246 | Endothelial cell-selective adhesion molecule |
| ENSG00000164251 | P55085 | F2RL1 GPR11 PAR2 | Proteinase-activated receptor 2 |
| ENSG00000131095 | P14136 | GFAP | Glial fibrillary acidic protein |
| ENSG00000176884 | Q05586 | GRIN1 NMDAR1 | Glutamate receptor ionotropic, NMDA 1 |
| ENSG00000173020 | P25098 | GRK2 ADRBK1 BARK BARK1 | Beta-adrenergic receptor kinase 1 |
| ENSG00000198873 | P34947 | GRK5 GPRK5 | G protein-coupled receptor kinase 5 |
| ENSG00000164082 | Q14416 | GRM2 GPRC1B MGLUR2 | Metabotropic glutamate receptor 2 |
| ENSG00000198822 | Q14832 | GRM3 GPRC1C MGLUR3 | Metabotropic glutamate receptor 3 |
| ENSG00000082701 | P49841 | GSK3B | Glycogen synthase kinase-3 beta |
| ENSG00000204632,  ENSG00000206506,  ENSG00000235346,  ENSG00000230413,  ENSG00000237216,  ENSG00000233095,  ENSG00000235680 | P17693 | HLA-G HLA-6.0 HLAG | HLA class I histocompatibility antigen, alpha chain G |
| ENSG00000163106 | O60760 | HPGDS GSTS PGDS PTGDS2 | Hematopoietic prostaglandin D synthase |
| ENSG00000119912 | P14735 | IDE | Insulin-degrading enzyme |
| ENSG00000131203 | P14902 | IDO1 IDO INDO | Indoleamine 2,3-dioxygenase 1 |
| ENSG00000111537 | P01579 | IFNG | Interferon gamma |
| ENSG00000136634 | P22301 | IL10 | Interleukin-10 |
| ENSG00000110324 | Q13651 | IL10RA IL10R | Interleukin-10 receptor subunit alpha |
| ENSG00000150782 | Q14116 | IL18 IGIF IL1F4 | Interleukin-18 |
| ENSG00000115008 | P01583 | IL1A IL1F1 | Interleukin-1 alpha |
| ENSG00000125538 | P01584 | IL1B IL1F2 | Interleukin-1 beta |
| ENSG00000164399 | P08700 | IL3 | Interleukin-3 |
| ENSG00000113520 | P05112 | IL4 | Interleukin-4 |
| ENSG00000136244 | P05231 | IL6 IFNB2 | Interleukin-6 |
| ENSG00000169896 | P11215 | ITGAM CD11B CR3A | Integrin alpha-M |
| ENSG00000177301 | P16389 | KCNA2 | Potassium voltage-gated channel subfamily A member 2 |
| ENSG00000058085 | Q13753 | LAMC2 LAMB2T LAMNB2 | Laminin subunit gamma-2 |
| ENSG00000086730 | Q9GZY6 | LAT2 LAB NTAL WBS15 WBSCR15 WBSCR5 HSPC046 | Linker for activation of T-cells family member 2 |
| ENSG00000104972 | Q8NHL6 | LILRB1 ILT2 LIR1 MIR7 | Leukocyte immunoglobulin-like receptor subfamily B member 1 |
| ENSG00000123384 | Q07954 | LRP1 A2MR APR | Prolow-density lipoprotein receptor-related protein 1 |
| ENSG00000154589 | Q9Y6Y9 | LY96 ESOP1 MD2 | Lymphocyte antigen 96 |
| ENSG00000112062 | Q16539 | MAPK14 CSBP CSBP1 CSBP2 CSPB1 MXI2 SAPK2A | Mitogen-activated protein kinase 14 |
| ENSG00000107643 | P45983 | MAPK8 JNK1 PRKM8 SAPK1 SAPK1C | Mitogen-activated protein kinase 8 |
| ENSG00000186868 | P10636 | MAPT MAPTL MTBT1 TAU | Microtubule-associated protein tau |
| ENSG00000240972 | P14174 | MIF GLIF MMIF | Macrophage migration inhibitory factor |
| ENSG00000196611 | P03956 | MMP1 CLG | Interstitial collagenase |
| ENSG00000149968 | P08254 | MMP3 STMY1 | Stromelysin-1 |
| ENSG00000100985 | P14780 | MMP9 CLG4B | Matrix metalloproteinase-9 |
| ENSG00000141052 | Q8IZQ8 | MYOCD MYCD | Myocardin |
| ENSG00000134259 | P01138 | NGF NGFB | Beta-nerve growth factor |
| ENSG00000089250 | P29475 | NOS1 | Nitric oxide synthase, brain |
| ENSG00000007171 | P35228 | NOS2 NOS2A | Nitric oxide synthase, inducible |
| ENSG00000164867 | P29474 | NOS3 | Nitric oxide synthase, endothelial |
| ENSG00000104967 | Q9UNW9 | NOVA2 ANOVA NOVA3 | RNA-binding protein Nova-2 |
| ENSG00000197822 | Q16625 | OCLN | Occludin |
| ENSG00000225553 | O43189 | PHF1 PCL1 | PHD finger protein 1 |
| ENSG00000132170 | P37231 | PPARG NR1C3 | Peroxisome proliferator-activated receptor gamma |
| ENSG00000080815 | P49768 | PSEN1 AD3 PS1 PSNL1 | Presenilin-1 |
| ENSG00000143801 | P49810 | PSEN2 AD4 PS2 PSNL2 STM2 | Presenilin-2 |
| ENSG00000107317 | P41222 | PTGDS PDS | Prostaglandin-H2 D-isomerase |
| ENSG00000073756 | P35354 | PTGS2 COX2 | Prostaglandin G/H synthase 2 |
| ENSG00000143546 | P05109 | S100A8 CAGA CFAG MRP8 | Protein S100-A8 |
| ENSG00000160307 | P04271 | S100B | Protein S100-B |
| ENSG00000196136 | P01011 | SERPINA3 AACT GIG24 GIG25 | Alpha-1-antichymotrypsin |
| ENSG00000174640 | Q92959 | SLCO2A1 OATP2A1 SLC21A2 | Solute carrier organic anion transporter family member 2A1 |
| ENSG00000145335 | P37840 | SNCA NACP PARK1 | Alpha-synuclein |
| ENSG00000157152 | Q92777 | SYN2 | Synapsin-2 |
| ENSG00000102003 | P08247 | SYP | Synaptophysin |
| ENSG00000136869 | O00206 | TLR4 | Toll-like receptor 4 |
| ENSG00000115297 | O43763 | TLX2 HOX11L1 NCX | T-cell leukemia homeobox protein 2 |
| ENSG00000232810,  ENSG00000206439,  ENSG00000228849,  ENSG00000204490,  ENSG00000223952,  ENSG00000230108,  ENSG00000228321 | P01375 | TNF TNFA TNFSF2 | Tumor necrosis factor |
| ENSG00000028137 | P20333 | TNFRSF1B TNFBR TNFR2 | Tumor necrosis factor receptor superfamily member 1B |
| ENSG00000121858 | P50591 | TNFSF10 APO2L TRAIL | Tumor necrosis factor ligand superfamily member 10 |
| ENSG00000101255 | Q96RU7 | TRIB3 C20orf97 NIPK SKIP3 TRB3 | Tribbles homolog 3 |
| ENSG00000100300 | P30536 | TSPO BZRP MBR | Translocator protein |
| ENSG00000100300 | B1AH88 | TSPO PBRS | Putative peripheral benzodiazepine receptor-related protein |
