# Supplementary material for: A Computational Exploration of the Molecular Network Associated to Neuroinflammation in Alzheimer’s Disease
Source: Front Pharmacol. 2021 Jul 15;12:630003. doi: 10.3389/fphar.2021.630003 (PMC8319636; doi:10.3389/fphar.2021.630003)
Supplement: Supplementary file 2 [file Table2.DOCX]

**Supplementary table 2. microRNA-protein interactions.**

| **Mature microRNA** | **Target interactions** |
| --- | --- |
| hsa-let-7a-5p | IL6,CXCL8 |
| hsa-let-7b-5p | TLR4,CXCL8 |
| hsa-let-7d-5p | CXCL8 |
| hsa-let-7e-5p | CXCL8 |
| hsa-let-7f-5p | IL6,CXCL8 |
| hsa-let-7g-5p | CXCL8 |
| hsa-let-7i-5p | TLR4,CXCL8 |
| hsa-mir-100-5p | AKT1 |
| hsa-mir-106a-5p | IL10,CXCL8,IL6 |
| hsa-mir-107 | IL6 |
| hsa-mir-10b-5p | AKT1 |
| hsa-mir-125a-5p | AKT1 |
| hsa-mir-125b-5p | AKT1,TNF |
| hsa-mir-126-3p | AKT1 |
| hsa-mir-1294 | CXCL8 |
| hsa-mir-130a-3p | TNF |
| hsa-mir-142-3p | IL6 |
| hsa-mir-143-3p | TNF,AKT1 |
| hsa-mir-146a-5p | IL6,TLR4,CXCL8 |
| hsa-mir-146b-5p | IL6,TLR4 |
| hsa-mir-155-5p | AKT1,CXCL8,IL6,CCL2,NOS3 |
| hsa-mir-17-5p | TNF |
| hsa-mir-185-3p | AKT1 |
| hsa-mir-185-5p | AKT1 |
| hsa-mir-192-5p | AKT1 |
| hsa-mir-194-5p | IL10 |
| hsa-mir-196b-5p | AKT1 |
| hsa-mir-199a-3p | AKT1 |
| hsa-mir-19a-3p | TNF,AKT1,IL10 |
| hsa-mir-204-5p | CXCL8 |
| hsa-mir-21-5p | TLR4 |
| hsa-mir-22-3p | AKT1 |
| hsa-mir-223-3p | IL6 |
| hsa-mir-23a-3p | CXCL8 |
| hsa-mir-24-3p | IL4,CCL2,NOS3,TNF |
| hsa-mir-26a-5p | IL6 |
| hsa-mir-26b-5p | TLR4,CCL2,AKT1 |
| hsa-mir-326 | AKT1 |
| hsa-mir-330-5p | AKT1 |
| hsa-mir-335-3p | NOS3 |
| hsa-mir-335-5p | IL6,CXCL8,TLR4,IL4,NOS3 |
| hsa-mir-340-5p | IL4,AKT1 |
| hsa-mir-365a-3p | AKT1,IL6 |
| hsa-mir-365b-3p | AKT1 |
| hsa-mir-374b-5p | AKT1 |
| hsa-mir-378a-3p | AKT1 |
| hsa-mir-378b | AKT1 |
| hsa-mir-378c | AKT1 |
| hsa-mir-378d | AKT1 |
| hsa-mir-378e | AKT1 |
| hsa-mir-378f | AKT1 |
| hsa-mir-378i | AKT1 |
| hsa-mir-409-3p | AKT1 |
| hsa-mir-451a | AKT1,IL6 |
| hsa-mir-4659a-3p | TLR4 |
| hsa-mir-4659b-3p | TLR4 |
| hsa-mir-4677-3p | CXCL8 |
| hsa-mir-4742-3p | AKT1,TLR4 |
| hsa-mir-625-3p | AKT1 |
| hsa-mir-654-3p | AKT1 |
| hsa-mir-7-5p | TLR4 |
| hsa-mir-93-5p | CXCL8 |
| hsa-mir-99a-5p | AKT1 |
