# Supplementary material for: A Computational Exploration of the Molecular Network Associated to Neuroinflammation in Alzheimer’s Disease
Source: Front Pharmacol. 2021 Jul 15;12:630003. doi: 10.3389/fphar.2021.630003 (PMC8319636; doi:10.3389/fphar.2021.630003)
Supplement: Supplementary file 3 [file Table3.DOCX]

**Supplementary table 3. lncRNA-protein interactions.**

| **lncRNA** | **Target interactions** | **Reference** |
| --- | --- | --- |
| GAS5 | IL10 | (1) |
| lnc-IL7R | IL6 | (2) |
| lncRNA-SRLR | IL6 | (3) |
| AFAP1-AS1 | AKT1 | (4) |
| HOTAIR | AKT1 | (5) |
| LINC00152 | AKT1 | (6) |
| MALAT1 | AKT1 | (7) |
| RMEL3 | AKT1 | (8) |
| UCA1 | AKT1 | (9) |
| IL1beta-eRNA | CXCL8 | (10) |
| IL1beta-RBT46 | CXCL8 | (10) |
| lnc-IL7R | CXCL8 | (2) |
| THRIL | TNF | (11) |
| lnc-DC | ITGAM | (12) |

1. Li Y, Li Y, Huang S, He K, Zhao M, Lin H, et al. Long non-coding RNA growth arrest specific transcript 5 acts as a tumour suppressor in colorectal cancer by inhibiting interleukin-10 and vascular endothelial growth factor expression. *Oncotarget* (2017) 8(8):13690-702. Epub 2017/01/19. doi: 10.18632/oncotarget.14625. PubMed PMID: 28099146; PubMed Central PMCID: PMCPMC5355130.

2. Cui H, Xie N, Tan Z, Banerjee S, Thannickal VJ, Abraham E, et al. The human long noncoding RNA lnc-IL7R regulates the inflammatory response. *Eur J Immunol* (2014) 44(7):2085-95. Epub 2014/04/12. doi: 10.1002/eji.201344126. PubMed PMID: 24723426; PubMed Central PMCID: PMCPMC4107034.

3. Xu Z, Yang F, Wei D, Liu B, Chen C, Bao Y, et al. Long noncoding RNA-SRLR elicits intrinsic sorafenib resistance via evoking IL-6/STAT3 axis in renal cell carcinoma. *Oncogene* (2017) 36(14):1965-77. Epub 2016/11/15. doi: 10.1038/onc.2016.356. PubMed PMID: 27841868.

4. Guo JQ, Li SJ, Guo GX. Long Noncoding RNA AFAP1-AS1 Promotes Cell Proliferation and Apoptosis of Gastric Cancer Cells via PTEN/p-AKT Pathway. *Dig Dis Sci* (2017) 62(8):2004-10. Epub 2017/04/30. doi: 10.1007/s10620-017-4584-0. PubMed PMID: 28451917.

5. Yu Y, Lv F, Liang D, Yang Q, Zhang B, Lin H, et al. HOTAIR may regulate proliferation, apoptosis, migration and invasion of MCF-7 cells through regulating the P53/Akt/JNK signaling pathway. *Biomed Pharmacother* (2017) 90:555-61. Epub 2017/04/14. doi: 10.1016/j.biopha.2017.03.054. PubMed PMID: 28407576.

6. Zhang Y, Xiang C, Wang Y, Duan Y, Liu C, Jin Y, et al. lncRNA LINC00152 knockdown had effects to suppress biological activity of lung cancer via EGFR/PI3K/AKT pathway. *Biomed Pharmacother* (2017) 94:644-51. Epub 2017/08/09. doi: 10.1016/j.biopha.2017.07.120. PubMed PMID: 28787699.

7. Dong Y, Liang G, Yuan B, Yang C, Gao R, Zhou X. MALAT1 promotes the proliferation and metastasis of osteosarcoma cells by activating the PI3K/Akt pathway. *Tumour Biol* (2015) 36(3):1477-86. Epub 2014/11/29. doi: 10.1007/s13277-014-2631-4. PubMed PMID: 25431257.

8. Goedert L, Pereira CG, Roszik J, Placa JR, Cardoso C, Chen G, et al. RMEL3, a novel BRAFV600E-associated long noncoding RNA, is required for MAPK and PI3K signaling in melanoma. *Oncotarget* (2016) 7(24):36711-8. Epub 2016/05/12. doi: 10.18632/oncotarget.9164. PubMed PMID: 27167340; PubMed Central PMCID: PMCPMC5095033.

9. Yang C, Li X, Wang Y, Zhao L, Chen W. Long non-coding RNA UCA1 regulated cell cycle distribution via CREB through PI3-K dependent pathway in bladder carcinoma cells. *Gene* (2012) 496(1):8-16. Epub 2012/01/31. doi: 10.1016/j.gene.2012.01.012. PubMed PMID: 22285928.

10. NE II, Heward JA, Roux B, Tsitsiou E, Fenwick PS, Lenzi L, et al. Long non-coding RNAs and enhancer RNAs regulate the lipopolysaccharide-induced inflammatory response in human monocytes. *Nat Commun* (2014) 5:3979. Epub 2014/06/10. doi: 10.1038/ncomms4979. PubMed PMID: 24909122; PubMed Central PMCID: PMCPMC4061460.

11. Li Z, Chao TC, Chang KY, Lin N, Patil VS, Shimizu C, et al. The long noncoding RNA THRIL regulates TNFalpha expression through its interaction with hnRNPL. *Proc Natl Acad Sci U S A* (2014) 111(3):1002-7. Epub 2013/12/29. doi: 10.1073/pnas.1313768111. PubMed PMID: 24371310; PubMed Central PMCID: PMCPMC3903238.

12. Wang P, Xue Y, Han Y, Lin L, Wu C, Xu S, et al. The STAT3-binding long noncoding RNA lnc-DC controls human dendritic cell differentiation. *Science* (2014) 344(6181):310-3. Epub 2014/04/20. doi: 10.1126/science.1251456. PubMed PMID: 24744378.
